# Reactivity of Gypsum-Based Materials Subjected to Thermal Load: Investigation of Reaction Mechanisms

**DOI:** 10.3390/ma13061427

**Published:** 2020-03-20

**Authors:** Felix Krause, Bernhard Renner, Frederik Coppens, Jan Dewanckele, Matthias Schwotzer

**Affiliations:** 1 Karlsruhe Institute of Technology (KIT), Institute of Functional Interfaces (IFG), 76344 Eggenstein-Leopoldshafen, Germany; felix.krause@kit.edu; 2 Saint-Gobain Formula GmbH, 37445 Walkenried, Germany; bernhard.renner@saint-gobain.com; 3 TESCAN XRE, 9052 Ghent, Belgium; frederik.coppens@tescan.com (F.C.); jan.dewanckele@tescan.com (J.D.)

**Keywords:** gypsum, hemihydrate, reaction mechanism, dynamic micro-CT, thermal stress, in situ experiment

## Abstract

The thermal stability of gypsum-based materials, and in this context, especially their long-term behavior, is the background of our current research activities. A comprehensive investigation program was compiled in which detailed examinations of various model materials exposed to thermal loads were carried out. The understanding of the partly not entirely consistent state of knowledge shall be sharpened especially by in situ observations of the thermally induced conversion reaction of gypsum into hemihydrate. The temporal course of the reaction was investigated non-destructively by in situ investigations in a high-resolution X-ray computed tomography setup, and the experiment was accompanied by detailed characterizations of the microstructure and composition. In this contribution, selected results of experiments with a high-purity natural gypsum rock as the model substance are presented. Studying the influence of temperature on the reaction showed that, even under supposedly dry conditions, the reaction could take place at much lower temperatures than usually reported in the literature. It was demonstrated that the transformation of gypsum into hemihydrate could take place at a temperature of already 50 °C. The results indicated that even under “classical” heating conditions in a conventional oven, the dissolution and crystallization processes in water films on the mineral surfaces could be suggested to be a driving force for the reaction. A corresponding reaction model, which took these aspects into account, was proposed in this work.

## 1. Introduction

The historical horizon of gypsum-related technology covers at least 11,000 years. The thermal behavior of gypsum (CaSO_4_ · 2 H_2_O) plays a central role in the production of gypsum-based binders and the range of applications of the materials. Even hundreds of years ago, the details of process control in binder production, such as the use of water vapor, were already documented and discussed as part of the state-of-the-art (e.g., [1]). The thermal stability of gypsum-based materials has always played an important role, even in historical times. Thus, Stark and Wicht [2] in their compilation of the history of gypsum emphasized the importance of gypsum-based materials in fire protection in the Middle Ages in Europe. Although the thermal behavior of gypsum-based systems has been important for numerous technologies for thousands of years and has always been the subject of scientific activities, the mechanisms of the processes that take place during the exposure of gypsum to elevated temperatures are still controversial, as stated and reviewed, e.g., in [3].

Numerous different models for the mechanism of the formation of calcium sulfate hemihydrate from gypsum (Equation (1)) are found in literature. It is commonly differentiated between a reaction taking place in aqueous solution or under atmospheric conditions [4]. While the reaction in aqueous solution is classically described in terms of solution equilibria, the mechanism of the atmospheric reaction is frequently understood as a solid-state reaction [5]. In this regard, the decomposition or dissociation, respectively, of gypsum [6] with the associated release of water (vapor) is sometimes assumed to be the starting point [7]. In line with this approach, the following steps of the solid-state reaction, which are assumed to build up the hemihydrate structure, are considered to be based on a topo-tactic nucleation (e.g., [8]). In this context, a reaction path involving several reaction stages with the products of differing water contents was specified and investigated (e.g., [3,9]). It seems to be accepted here that first of all, an anhydrous “hemihydrate” structure is built up, γ-CaSO_4_, which is then filled up according to the availability of water to a maximum water content that is adapted to the physical conditions, such as relative humidity and temperature (e.g., [3,10]). Therefore, by changing these conditions, substances with variable water contents can be formed. The supposedly most stable form is the so-called hemihydrate with a molar water content of 0.5, which is known as the mineral bassanite [4]. For compounds of this system, a maximum water content of 0.8 has been described in the literature [11]. The various possible “hydrate states” are closely related to the crystal structure of the respective substance [10,12].

(1) $$CaSO_{4}\cdot 2\;H_{2}O\;\overset{\Delta T}{\to }\;\gamma -CaSO_{4}+2\;H_{2}O↑\;\overset{\Delta T;\;p\left(H_{2}O\right)}{\to }\;CaSO_{4}\cdot n\;H_{2}O+\left(2-n\right)H_{2}O↑\;with\;n\leq 0.8$$

The temperature conditions at which “wet-chemical” processes take place and thus with the temperature dependencies of the solubility equilibria in the aqueous milieu have been described in good detail (e.g., summarized in [4]). On the other hand, there is no generally accepted consensus on the relevant temperatures for a reaction under ambient conditions, e.g., for various thermo-analytical processes. The temperatures commonly mentioned in the various studies are in the range of 100 °C (e.g., [5,8,9]). However, there are also occasional indications that temperatures well below 100 °C (e.g., [8,9,13]) could also be relevant for the conversion of gypsum into hemihydrate, which, however, other authors negated [14]. Concerning transformation temperatures below 100 °C, reaction mechanisms are also discussed, which add aspects to the above-mentioned viewpoints. For example, in [13], the potential role of processes such as early “melt formation” at 38 °C was discussed. In any case, the particular role of water vapor is pointed out here, which is of relevance for the kinetics of the reaction (e.g., [15,16]).

Since the detailed analysis of the evolvement of water vapor and its transport in the porous materials is usually the main focus of the diverse studies regarding fire protection, the reaction mechanism of the thermally-induced formation of hemihydrate is commonly considered as a “dehydration reaction”, i.e., solid state reaction (e.g., [17]). A widely shared view of the role of water vapor is that steam acts as an inhibitor for the “dehydration reaction” of gypsum, as van der Heijden et al. summarized [18]. Furthermore, thermodynamic studies also show that the stability of the hemihydrate in the phase diagrams obtained is also associated with temperatures clearly below 100 °C (e.g., [4,19]). This circumstance and also the many variants of both the stated transformation temperatures and the numerous outlines of an underlying reaction mechanism indicate that—despite the efforts made so far to investigate the dehydration paths of gypsum—the system is still far from being well understood.

In view of this, a comprehensive research project was initiated to provide an appropriate complement to the knowledge currently available. In this context, the research activities focused on the reaction mechanisms of the processes occurring in gypsum-based materials during thermal load. These finally resulted in material changes, affected in turn their properties, and—with regard to the manifold technical applications—their performance. To this aim, systematic investigations on the thermal behavior were carried out on various model materials ranging from polycrystalline systems, as gypsum-bound materials, as well as natural gypsum rocks, to single crystals. In this contribution, selected results of the investigations on the thermal behavior of a natural gypsum rock are presented. These findings will be applied to discuss the relationships between the temporal development of mineralogy, the thermal state, as well as the microstructure of the material. Therefore, time-resolved tomographic investigations are performed and supplemented by a set of material characterizations, as well as regarding the thermal diffusivity in the material. An innovative high-resolution X-ray computed tomography technology is used to follow these dynamic processes closely. Furthermore, additional detailed investigations about the reaction characteristic as a function of temperature are carried out to obtain further insight into the mechanism of the reaction.

## 2. Materials and Methods

### 2.1. Materials Used

The experiments were carried out with a natural gypsum rock from the Zechstein formations of the southern Harz Mountains, Germany. For the respective investigations, drill cores were taken from the rock or powder samples were produced. The rock was a high-purity gypsum rock (gypsum content >98 wt.%; accessory components were dolomite (Ca,Mg)CO_3_, celestine SrSO_4_, and quartz SiO_2_. The fundamental characterization of the mineralogical composition of gypsum rock, which was used as the sample material for the further exposure experiments in this study, was carried out using X-ray diffraction (XRD). The quantification of the gypsum content was performed by thermogravimetry (TGA). Accessory compounds of the rock were identified in electron microscopic investigations by means of energy dispersive X-ray spectrometry (EDS).

### 2.2. Experimental and Analytical Methods

For this study, experiments were carried out with compact samples cut or drilled out of the rock, as well as with powder samples. Table 1 illustrates the assignment of the investigation methods to the respective samples.

**Figure 1:**
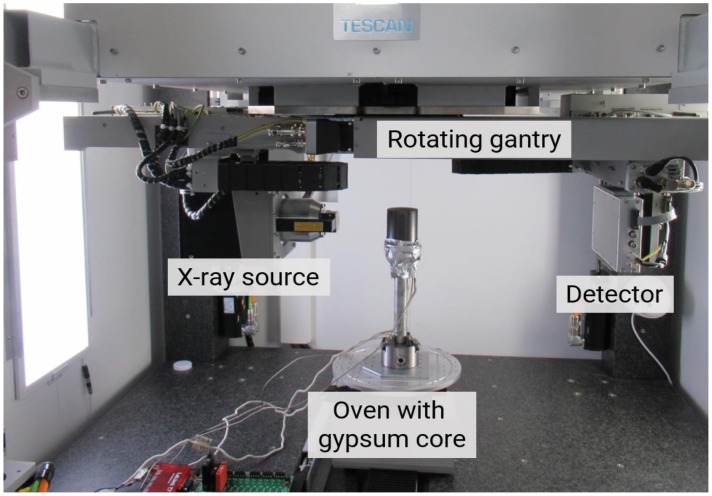
Oven with gypsum core samples mounted in the TESCAN DynaTOM, a horizontal gantry-based micro-CT system.

To determine the microstructural changes in the gypsum rock during the exposure experiments, high-resolution X-ray computed tomography investigations (micro-CT) were performed. The micro-CT investigations were performed with a TESCAN DynaTOM system (TESCAN, Brno, Czech Republic) [20]. Figure 1 illustrates the setup. DynaTOM’s unique horizontal gantry-based hardware design, with a source and detector rotating around a stationary sample, enabled continuous scanning without compromising in situ rig flexibility. This made it feasible to install experimental setups in the device allowing performing in situ dynamic characterization of material changes. A core of the gypsum rock (diameter 1.5 cm, height 1.3 cm) was inserted in an oven and exposed to a temperature of 100 °C. A continuous acquisition was performed during exposure of the gypsum core to the elevated temperature. Afterwards, the data were reconstructed, and differential images were obtained with the first scan (t = 0) of the time series as the reference volume. In total, 12 acquisitions were taken at time interval of 2 h. The voxel size of these scans was 32.6 µm.

Accompanying the in situ investigations with the micro-CT, the temperature development in a gypsum core with identical dimensions was determined in a conventional drying oven. Thermocouples (Pt100) were placed in the center of the core and in its near-surface area. For this purpose, holes were drilled into the cores, and the thermocouples were inserted. The boreholes were sealed afterwards with a plaster binder. As a reference, the furnace temperature was recorded with another thermocouple. The data acquisition was carried out with a multi-channel measuring electronics Spider 8 from Hottinger Baldwing Messtechnik, Darmstadt, Germany.

The additional characterization of the pore structure was carried out using mercury intrusion porosimetry (MIP). The instrument combination Pascal 140 EVO and Pascal 440 EVO from Thermo Fisher Scientific was employed for this analysis. The gypsum samples were dried in a drying oven at 40 °C until weight constancy was achieved.

Electron microscopic examinations were performed with a Vega III from TESCAN, Brno, Czech Republic, and a FEG-ESEM Quattro from Thermo Fischer Scientific, Eindhoven, Netherlands, equipped with an Octane Elite EDS system from EDAX, Mahwah, USA. Prior to the electron microscopic investigations, the samples were coated with approximately 4 nm platinum. Microstructural and petrographic examinations of the sample rock were carried out on thin section specimens using a polarization microscope type DM 4 P from Leica, Wetzlar, Germany.

The mineralogical composition was investigated by X-ray diffraction (XRD) on powder samples with a D8 Advance diffractometer from Bruker AXS, Knielingen, Germany. For these investigations, Cu_Kα1,2_ X-rays were used (wavelength: 1.5418 Å). Thermogravimetric investigations on powder samples were performed with a TGA2 from Mettler Toledo, Greifensee, Switzerland. All measurements were carried out under a N_2_ atmosphere. For these two types of investigations, sample material from the gypsum rock was pulverized with an agate mortar.

A laser flash analysis (LFA) was used to determine the thermal diffusivity, a thermophysical parameter that represents the rate at which a temperature change passes through a material. The thermal diffusivity is for polycrystalline materials a function of temperature. For the gypsum rocks examined here, this was determined over a temperature range from −100 °C to 200 °C. In this experiment, rectangular samples were used (edge length 1 cm, height 1 mm). The measurements were carried out with the LFA 457 type from Netzsch, Selb, Germany.

## 3. Results

### 3.1. Thermal Behavior of the Rock Sample

**Figure 2:**
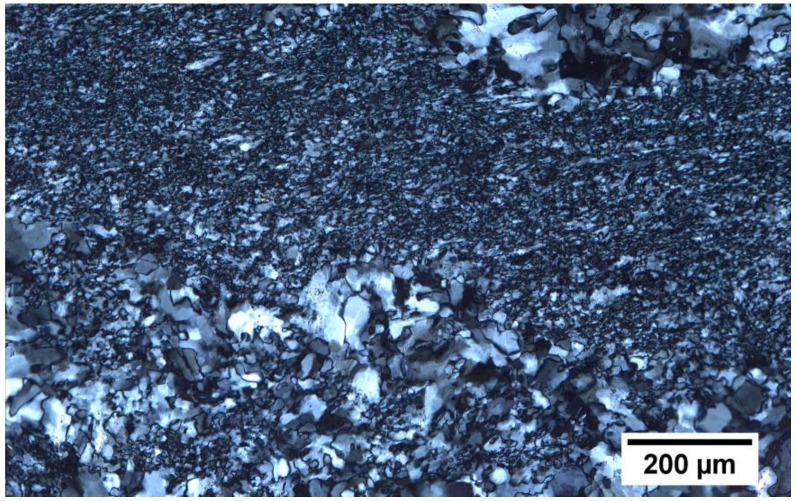
Microscopic image of the microstructure of a thin section specimen of the gypsum rock used in the experiments (recorded with cross-polarization).

The microstructure of the gypsum rock from the Zechstein formations of the southern Harz Mountains, in northern Germany, was examined with a polarization microscope (Figure 2). The grain size of the rock sample ranged from a few µm up to several tens of µm.

The predominant part of the gypsum grains was however very fine-grained. Some coarser grained areas were embedded in the fine-grained matrix. However, the larger grains were typically divided into numerous sub-grains. In the thin section, almost exclusively, gypsum crystals could be seen (apart from a few accessory minerals, e.g., carbonates). It showed a dense rock structure, in which no noticeable pores were visible under the microscope.

**Figure 3:**
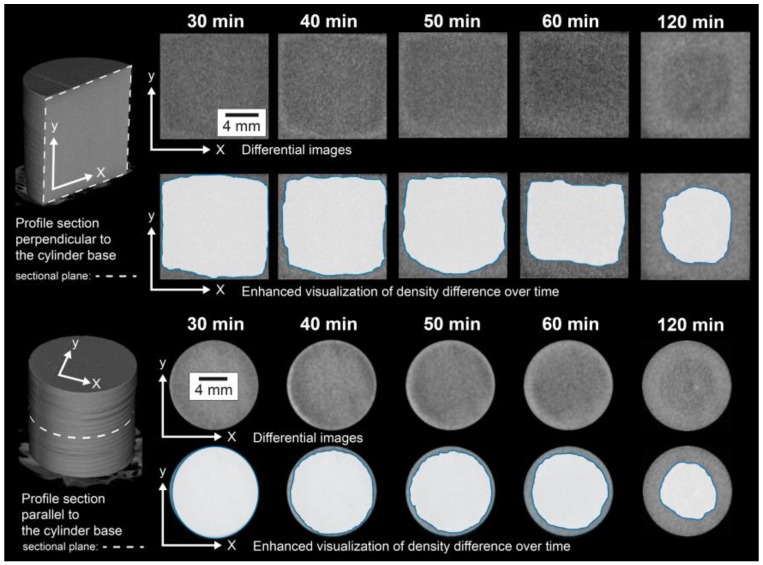
Results of the in situ micro-CT examinations in cross-sections perpendicular and parallel to the base of the core over time (for 2 h at 100 °C).

The changes generated in a gypsum rock when subjected to thermal load at 100 °C were recorded directly by micro-CT. The measured results are shown in Figure 3. Cross-sections through the core are shown perpendicular (upper part of the image) and parallel (lower part of the image) to the base of the cylinder. By dividing the first volume (t = 0) by the subsequent volumes, changes in attenuation of the X-rays and, thus, information regarding the relative density became visible. The differential images in the upper row of each figure show the local distribution of the attenuation of the X-rays at different times of the experiment. In this differential representation, areas that caused a weaker attenuation of the X-rays appeared darker than areas that attenuated the radiation to a greater extent. For a better visualization of this observation, this area is brightly outlined and thereby graphically highlighted in a series of images shown directly below the corresponding images of the differential data. The temporal development during the first 60 min at 100 °C and a final state after 120 min is shown. It could be observed that during the exposure of the core to high temperatures, areas developed in the gypsum rock that showed different attenuation of the X-rays. Towards the center of the sample, a darker zone appeared after the process time, which was supposed to present a lower density than the peripheral area. These observations could be made for the two different sections shown in Figure 3.

**Figure 4:**
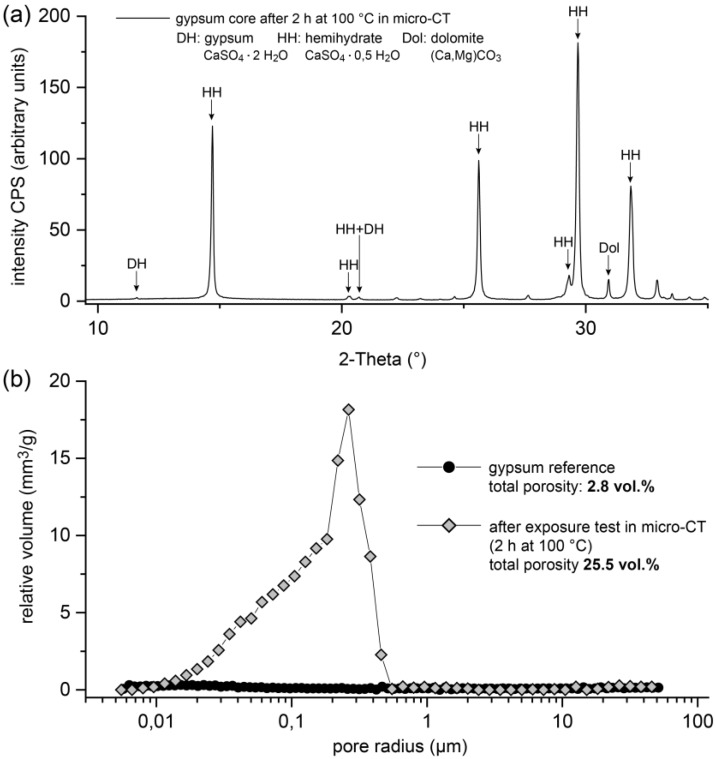
XRD measurement (**a**) and results of the investigation of the pore structure by mercury intrusion porosimetry (MIP) (**b**) of the sample directly after micro-CT tests (2 h at 100 °C). The MIP results are contrasted with the measurement of an untreated reference sample.

By means of X-ray diffraction, it was shown that the drill core consisted almost exclusively of hemihydrate after the experiment (Figure 4a). Carbonate minerals such as dolomite were identified as a minor component. Only traces of gypsum were detectable.

Figure 4b shows the results of the investigation of the pore structure using MIP. The total porosity of the material was 25.5 vol.%. The detected pore sizes ranged from 0.02 to 0.5 µm. A pronounced maximum in the distribution of the pore sizes was found for pore radii of approximately 0.2 µm. In contrast, the measurements of an untreated rock sample showed a porosity of <3 vol.%. In this reference measurement, a noticeable maximum in the distribution of the pore radii was not observed.

**Figure 5:**
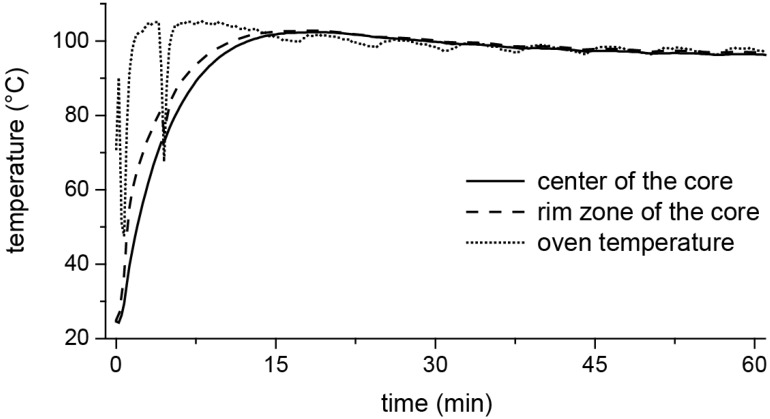
Temporal evolution of the temperature of a core of the same dimensions as micro-CT experiments from the gypsum rock in an oven at 100 °C.

The temporal evolution of the temperature in the gypsum rock core that was exposed in a drying oven at 100 °C is shown in Figure 5. In this setup, the rock sample achieved the oven temperature after 15 min. Here, the oven temperature was reached later in the center of the sample than in its peripheral zones.

**Figure 6:**
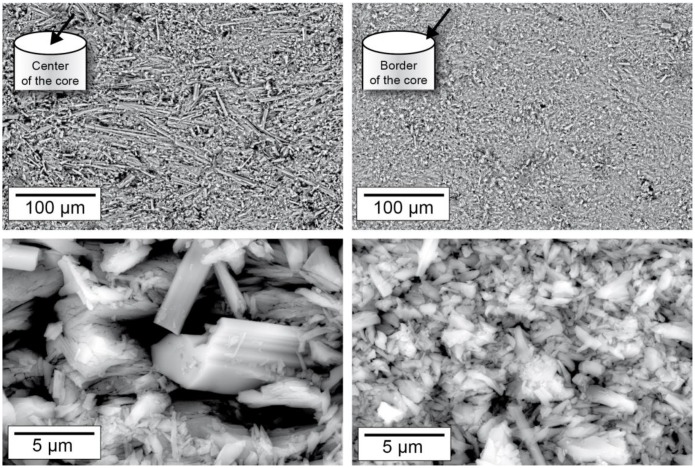
Electron microscopic examination of the microstructure of the gypsum sample after the exposure experiment (100 °C, 2 h) at the edge of the core and in its center.

The results of the examination of the microstructure by electron microscopy are shown in Figure 6. The images were recorded on the top face of a cylindrical disk, which was taken from the sample core after the in situ experiment in the middle (shown schematically in the upper left corner of the image). The left-hand images were taken in the middle of the disc; the right-hand images were taken at the edge of the sample. The lower part of the illustration shows a detail enlargement of the area shown above. In both areas, the material was composed of stem-like or needle-shaped crystals, which often showed well-developed crystal faces. Nevertheless, the two areas differed significantly in their microstructure. The microstructure of the edge region (Figure 6, right) appeared much finer grained compared to the inner area (Figure 6, left). It was predominantly made up of columnar or compact particles of dimensions of approximately 1-10 µm. In contrast, the microstructure in the area near the center of the sample was composed mainly of large needle-shaped crystals. These crystals featured a length of up to 100 µm. Spaces between the particles appeared as dark areas in these images. As the higher magnification images showed (Figure 6, bottom), the inner area of the sample, which as mainly composed of the needle-shaped crystals, had larger gaps, i.e., pores.

**Figure 7:**
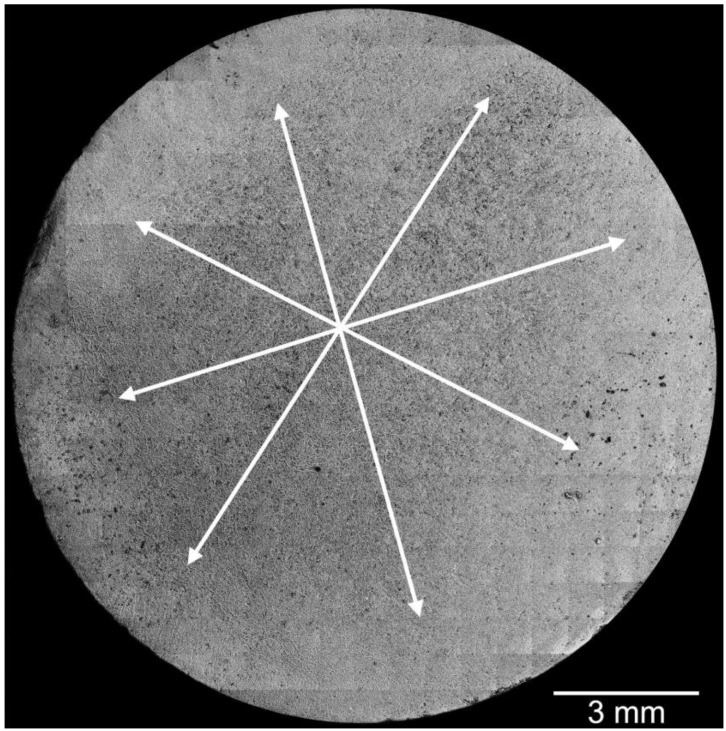
Compiled overview image from approximately 500 backscattered electron images of a profile section in the middle of the sample after the in situ experiment in the furnace in the micro-CT setup.

Grayscale levels and brightness values of backscattered electron images are widely used in the literature for the quantitative description of the pore structure of porous mineral systems based on image segmentation (e.g., [21]). Figure 7 shows a high-resolution overview image, composed of about 500 backscattered electron images, of a profile section in the middle of the sample after the in situ experiment in the oven in the micro-CT setup. In the inner region of the stitched image, an area was visible, which appeared significantly darker. This area in Figure 7 is coarsely marked by white arrows. A darker coloration of an image area of such an overview image was the result of the summation of the dark appearing gaps between the mineral particles (Figure 6). These differences in the brightness of the gray levels were an indicator of local differences in the pore structure. For the present case, it could therefore be assumed that the darker, inner area of the cross-sectional surface had a higher porosity (or at least a significantly higher fraction of large pores) than the lighter edge area.

### 3.2. Investigation of the Thermal Diffusivity as a Function of Temperature

**Figure 8:**
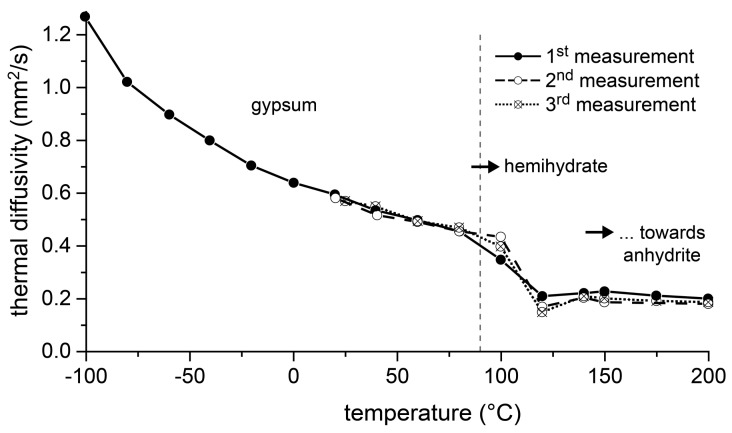
Measurement of the thermal diffusivity of the gypsum rock (as triple determination) by means of laser flash analysis (LFA).

The thermal diffusivity of a material is a measure of how quickly a temperature change in a material propagates and, thus, in turn, how quickly its temperature adapts to the ambient temperature. Figure 8 shows the results of measuring the thermal diffusivity of the gypsum rock. The investigation was carried out in the temperature interval from −100 °C to 200 °C. In the range from 20 °C to 200 °C two repeat measurements were performed. The results showed in the temperature range <100 °C a typical behavior for dense, polycrystalline rocks, in which the mineral grains are directly adjacent to each other without a gap. The rate of heat transport through such a material decreased with increasing temperature (e.g., [22]). The thermal diffusivity was reduced to more than 50% in the temperature range from −100 °C to 100 °C. When the temperature of the sample approached the range of 80 °C to 100 °C, the thermal diffusivity dropped again abruptly. At temperatures >120 °C, it remained almost constant with a value of approximately 0.2 mm²/s to 200 °C, which was less than a fifth of the value at −100 °C. This behavior was confirmed in the two repeat measurements.

### 3.3. Isothermal Treatment of Powder Samples

**Figure 9:**
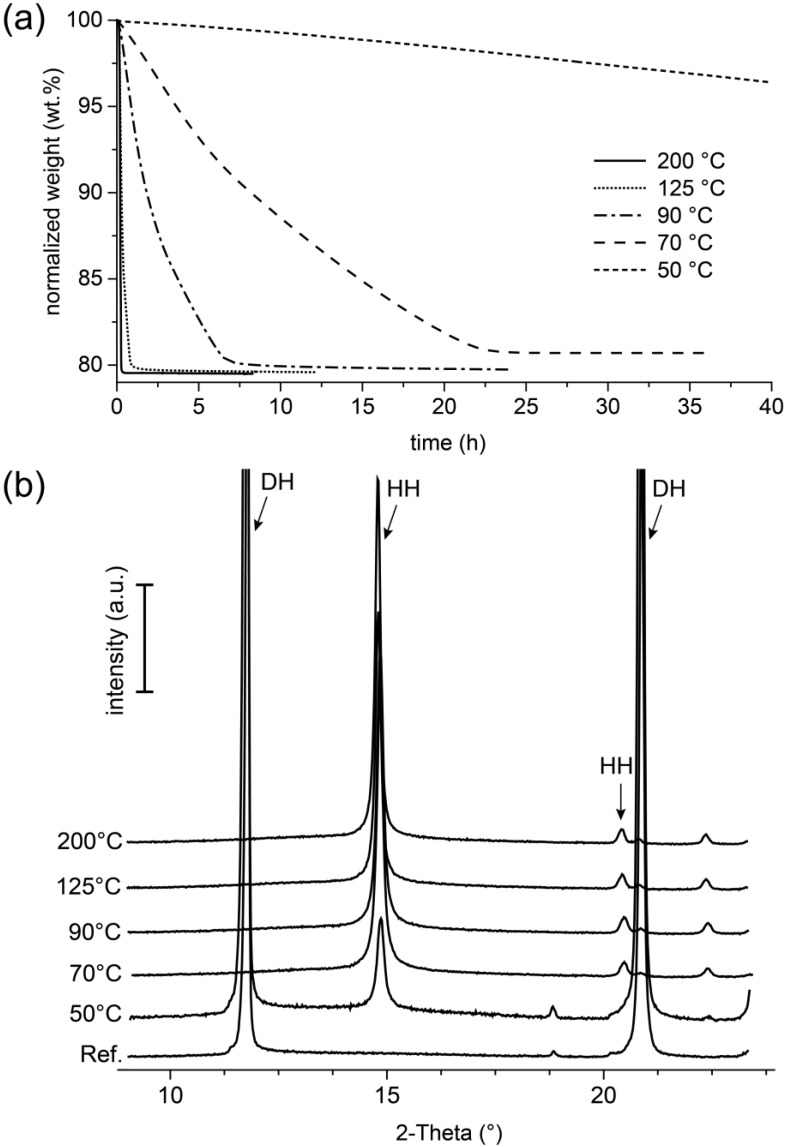
Results of the isothermal thermo-gravimetric investigations at 50 °C, 70 °C, 90 °C, 125 °C, and 200 °C (**a**) and the respective mineralogical characterization of the reaction products by means of XRD (**b**) (DH: gypsum, HH: hemihydrate, a.u.: arbitrary units).

For the investigation of the role of temperature in the transformation of gypsum into hemihydrate, powder samples of the gypsum rock were exposed in the TGA under isothermal conditions. The measurement was performed in an alumina ceramic crucible with a small hole in the cap. During the measurement, the measuring cell was continuously purged with nitrogen (20 mL/min). The temporal development of the weight of the samples (normalized to the initial weight, for all samples 35 mg ± 4 mg) at the respective set temperatures (50 °C, 70 °C, 90 °C, 125 °C, and 200 °C) is shown in Figure 9a. The measurements of the samples taken at 70 °C, 90 °C, 125 °C, and 200 °C were stopped after reaching a constant weight. The measurement, which was carried out at a temperature of 50 °C, was stopped after 40 h of exposure. In all cases, weight loss was detected over the duration of the test. The samples, which were exposed at 200 °C and 125 °C, reached a residual weight of 79.5 wt.% and 79.6 wt.% after approximately 20 min and 50 min, respectively. The distinct and apparently steady weight loss seemed to end in the experiments carried out at 90 °C and 70 °C after eight hours at a residual weight of 79.7 wt.% and 25 h at 80.7 wt.%, respectively. The experiment conducted at 50 °C was stopped when, after a duration of 40 h, the sample showed a weight loss of about 4 wt.%.

The mineralogical composition of the sample material from these experiments was analyzed by means of XRD after the measurement. The results are summarized in Figure 9b and compared with the measurement of the untreated material as a reference. No reflex could be detected in the samples measured at 70 °C, 90 °C, 125 °C, and 200 °C, which could be attributed to the mineral gypsum. Instead, prominent peaks of hemihydrate were detected. In comparison, the sample treated at 50 °C also showed distinct gypsum reflexes, but also, significant reflexes of hemihydrate were recorded.

## 4. Discussion

The thermal behavior of gypsum-based materials, as well as natural gypsum rocks, is of considerable importance for a variety of technical fields. Numerous examples can be found in the applications of these materials for fire protection in buildings, as well as in a number of industrial production processes. The long-term stability of gypsum-based materials in the construction industry, and thus their quality and performance, is closely related to their resistance against thermal loads. The thermal behavior of natural gypsum rocks is also relevant, for example, with regard to the durability of historical buildings, where such materials have been used in many cases. All these are examples of polycrystalline materials that undergo chemical-mineralogical changes under the influence of elevated temperatures. However, up to the present time—as explained at the beginning with a short summary of the current scientific discussion (e.g., [3,4,5,6,7,8,9,10,11,12,13,14,15,16,17,18,19,20,21,22,23])—the understanding of the mechanisms underlying this conversion reaction is still incomplete and lacks consistency. This is particularly true for the related thermodynamic data basis, e.g., the transformation temperatures in the system CaSO_4_-H_2_O, as summarized in [23]. Thus, in this contribution, selected aspects of a research project shall sharpen the view on the processes that occur in these materials when subjected to thermal loads.

It is often assumed that a temperature increase leads to a transformation of the hydrate mineral gypsum (CaSO_4_ × 2 H_2_O), first into the anhydrous γ-CaSO_4_ phase with the hemihydrate structure, which then takes up water into its structure according to the ambient conditions given by the atmosphere (e.g., [8,12]). This is also confirmed by the results of the thermogravimetric investigations of this study shown in Figure 9a, in which a loss of weight was detected after thermal stress, which represented with about 20 wt.% an almost complete release of the crystal water in the gypsum. However, these results also showed that the final weight after the experiment was related to the reaction temperatures in the TGA. The fact of the loss of mass, which at lower reaction temperatures tended to be lower after the end of the measurement than in the experiments at higher temperatures, indicated that phases with equivalent crystal structures (Figure 9b), but different water contents formed here.

To study the effects of thermal loads in polycrystalline systems, a high-purity natural gypsum rock with a particularly dense and fine-grained structure (Figure 2) was used as the model substance in the experiments presented here. In an in situ investigation, the rock was exposed to a temperature of 100 °C in a furnace for 120 min, and the resulting material changes were continuously recorded by means of micro-CT. The micro-CT detected differences in the attenuation of the X-rays by the sample. Usually, materials can be contrasted that show significant differences in X-ray attenuation. In a very homogeneously composed material, such as the present gypsum rock, such differences were not present. Individual grains of gypsum could therefore hardly be distinguished. Moreover, the maximum achievable resolutions were in the order of micrometers, which also made it almost impossible to differentiate individual mineral grains in this type of rock. However, during the exposure experiment at 100 °C, significant differences in the attenuation of the X-rays and thus in the density were observed. After 120 min, a region of lower density than the peripheral region was formed in the interior part of the sample (Figure 3). In principle, this difference could also be caused by differing contents of gypsum and hemihydrate. However, this difference was not due to differences in the mineralogical composition. This was confirmed by the examination of the mineralogical composition (XRD), which showed that the gypsum in the core was transformed almost completely into hemihydrate (Figure 4a). Therefore, these differences must consequently result from the microstructure.

Since the material was completely transformed, the total porosity over the entire area of the sample should be comparable. The total porosity of approximately 25.5 vol.% of the material after the experiment (Figure 4b) corresponded in good approximation to a theoretical porosity value of 26.3 vol.%, which could be calculated from the molar volumes of gypsum and hemihydrate if gypsum (assuming a constant volume) was completely converted into hemihydrate. Nonetheless, electron microscopic examination revealed local differences in the microstructure between the outer area, which appeared denser in micro-CT examination, and the inner area. The inner area tended to have larger pores, and the hemihydrate crystals were significantly larger than in the peripheral area (as shown in Figure 6). However, it could be assumed that the different pore sizes, some of which were significantly smaller than the resolution of the micro-CT scan, contributed potentially in different ways to the micro-CT measurement results. The difference in density determined by micro-CT could therefore as well be due to a tendency towards a higher proportion of large pores. This was also supported by the high-resolution overview image obtained by electron microscopy using the backscattered electron detector (Figure 7). The darker appearing area shown here had similar dimensions as the corresponding area in the micro-CT image (Figure 3). However, on the basis of the investigations, it could not be fully verified whether these variations were only due to differences in the pore size distribution or whether light and dark areas also differed in terms of total porosity.

Thus, one conclusion could be reliably drawn from the results: in this experiment, the transformation of gypsum into hemihydrate occurred under different conditions inside the core than in its peripheral areas. 

In terms of the total experiment duration, the temperature that was present in the sample was quickly equilibrated with the furnace temperature over the entire sample area (after approximately 15 min, as shown in Figure 5). Although the temperature conditions were comparable throughout the volume of the sample, the dimensions of the particles of the reaction products—in the inner area with the larger hemihydrate crystals compared to the fine-grained peripheral area—differed drastically and, thus, indicated differing reaction regimes. This implied that the transformation cannot be described unreservedly by the idea of a pure solid state or a “dehydration” reaction, respectively. The fact that the grain size of the large hemihydrate crystals in the inner region of the material (Figure 6) was larger than the particle sizes of the original, untreated rock (Figure 2) also pointed towards the fact that these crystals must have been formed by crystallization processes from a solution.

A further indication of this could be obtained from the results of the isothermal thermogravimetry experiment carried out with the gypsum powder. Here, it was clearly demonstrated that the formation of hemihydrate from gypsum could take place at temperatures as low as 50 °C (Figure 9). With increasing temperature (definitely for the temperature interval of 50 °C to 200 °C), the rate of the transformation reaction increased as well. With regard to the in situ experiment, temperature conditions prevailed everywhere in the sample, which with regard to the solubility relationships in the CaSO_4_–H_2_O system, would allow dissolution of gypsum and subsequent crystallization of hemihydrate. According to literature data compiled in [24], this could for example be the case in the temperature interval from 79 °C to 109 °C. In contrast, Abriel et al. [8] described in their experiments the occurrence of hemihydrate at temperatures starting at approximately 57 °C. Against the background of the isothermal thermogravimetry experiments carried out here with the gypsum powder, in which the conversion was already observed at 50 °C, but the reaction rate with only a formation rate of hemihydrate of only 4 wt.% in 40 h, it must be concluded that the particular experimental conditions had a substantial influence on the temperature indicated in the individual study. The kinetics, as well as the presence of water seemed to be crucial factors here. However, it appeared that the common solubility data (e.g., [24,25], summarized e.g., in [4]), which were also the diverse basis for the considerations in recent studies, could not be fully used to derive a reaction mechanism. This was supported, for example, by the recently published surprising results of the work of Van Driesche et al. [26] and He et al. [27] in which it was described for the crystallization of gypsum from aqueous solutions—under conditions for which it is generally assumed that gypsum is the stable phase—that the growth of gypsum was preceded by a hemihydrate phase as the precursor.

**Figure 10:**
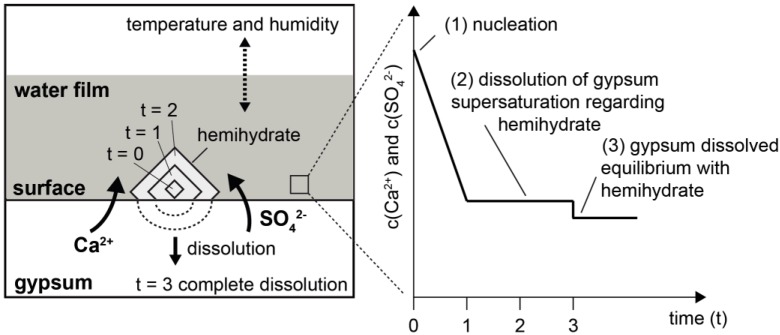
Model for the mechanism of the conversion of gypsum into hemihydrate.

These results and statements, particularly with reference to the reaction temperature, basically suggested that the thermally induced conversion of gypsum into hemihydrate could be regarded as a dissolution and crystallization reaction in an aqueous environment. However, this required that a transport process could take place in a solvent. A proposal for a reaction model is shown in Figure 10. The crucial element here was the availability of liquid water on the surfaces and/or grain boundaries of the gypsum crystals that constituted the rock. As a result of the dissolution of gypsum in an aqueous phase on its surface, conditions could arise there which led to the formation of stable hemihydrate nuclei (Figure 10, right, State 1). Their spontaneous further growth reduced the concentration of dissolved Ca^2+^ and SO_4_^2-^ in the vicinity of the gypsum-water interface and thus enforced the further dissolution of gypsum (Figure 10, right, State 2). Assuming that the physical parameters (in particular the temperature) remained stable, this process could continue until the gypsum was consumed or until an equilibrium was reached (Figure 10, right, State 3). Furthermore, the temperature not only determined the transformation of gypsum into hemihydrate through its influence on chemical solution equilibria, but also—along with the humidity—the volume of water present on the mineral surfaces. This is illustrated by the dotted arrow above the water film in the schematic representation of the model (Figure 10).

As a consequence of the increase in temperature in the gypsum rock, liquid water may be released, which in principle could already occur at temperatures below 100 °C (e.g., in [8] or [17]). It is a recognized fact that the presence of water films on the surfaces of porous gypsum-based systems plays a major role in many material properties (e.g., [28,29]) and is increasingly becoming the focus of attention as the driving force of solution and crystallization reactions [30]. The formation of water films or the accumulation of larger quantities of water with chemical properties comparable to those of liquid water can be obtained by a kind of condensation process. Such processes are closely related to the particle size of the mineral substrate. Recently, it has been stated that in particular, micron-sized particles (with a specific surface area of less than ~10 m^2^/g) should tend to be more capable of promoting such water condensation than submicron-sized particles [31].

If, with regard to the solubility equilibria, temperature conditions are realized according to which gypsum is less stable than hemihydrate, it is conceivable that sufficient supersaturation with regard to hemihydrate will occur in a water film on the surfaces in order to enable the nucleation of hemihydrate. If spontaneous growth of a nucleus continues, further dissolution of gypsum is forced until an equilibrium situation is reached. When such a process starts in pores or at grain boundaries, a new pore space will develop during this reaction due to the different volume requirements of hemihydrate and gypsum. The formation of a porous structure in the originally dense gypsum rock will result in a reduction of the rate at which thermal energy is introduced, as shown by the investigations of thermal diffusivity (Figure 8).

At this point, the hypothesis should be formulated that the available amount of water—which is dependent on temperature and humidity—controls the supersaturation situation. In this way, it could be explained that the hemihydrate is more fine-grained crystallized in the peripheral area, since the characteristic of supersaturation determines the particle size distribution of the crystals growing under the corresponding conditions [32]. One approach to explain this could be that drying can be realized faster here than inside the sample, so that crystallization from higher supersaturation could occur here due to the lower amount of water. Corresponding conclusions, especially regarding the role of transport and thus the availability of water for the temporal development of the reaction, were also drawn in [33]. The resulting increased nucleation rate could lead to a rapid growth of numerous small crystals. In deeper regions, larger crystals would be able to form following this model conception. Following this model, for the thermally induced conversion of gypsum into hemihydrate, not only the temperature, but also the moisture state is of substantial importance. This emphasizes the relevance of the drying regime—which porous gypsum-based materials are exposed to during thermal stress—for the thermal stability of the material.

## 5. Concluding Remarks

The results of this study indicated that the mechanism for the thermally induced conversion of gypsum into hemihydrate could be regarded as solution-crystallization chemistry even under “dry” conditions, e.g., in a conventional oven. It was postulated that this conversion chemistry occurred in water films on the mineral surfaces, which would develop in accordance with the specific physico-chemical boundary conditions. At this point, it must be clearly emphasized that the formation of hemihydrate could also occur at temperatures well below 100 °C, e.g., at 50 °C, as shown in this study. In this context, temperature was on the one hand a central factor for the chemical equilibria that controlled the solution and crystallization processes. Apparently, there was a close connection with the availability of water in the system. On the other hand, temperature was responsible, along with humidity, for maintaining this milieu on the surfaces, in which reactions and transport processes could take place. This moisture status and thus the drying regime should therefore have a significant influence on the extent to which a thermally induced transformation—according to this model, a more appropriate term than “thermal dehydration”—was realized and should be of comparable importance to temperature in this respect. Thus, the transport of both thermal energy and water were of crucial significance for the reaction. Furthermore, a more detailed consideration of this specific solubility chemistry in water films on mineral surfaces seems to be indispensable. In particular, with regard to reliable assessments of the material behavior of gypsum-bound systems, it appears promising to take an intensified look at the surface chemistry of the relevant phases in the system CaSO_4_–H_2_O.

## Figures and Tables

**Table 1:** Assignment of the methods to the respective samples in the exposure experiments.

Sample	Method
gypsum rock specimens	polarization microscopy, in situ X-ray computed tomography, temporal development of the sample temperature, X-ray diffraction, mercury intrusion porosimetry, electron microscopy, laser flash analysis.
gypsum powder	isothermal thermogravimetric analysis, X-ray diffraction.
